# *Madurella* real-time PCR, a novel approach for eumycetoma diagnosis

**DOI:** 10.1371/journal.pntd.0007845

**Published:** 2020-01-15

**Authors:** Amir Arastehfar, Wilson Lim, Farnaz Daneshnia, Wendy W. J. van de Sande, Ahmed H. Fahal, Marie Desnos-Ollivier, Gerrit S. de Hoog, Teun Boekhout, Sarah. A. Ahmed

**Affiliations:** 1 Westerdijk Fungal Biodiversity Institute, Utrecht, The Netherlands; 2 Erasmus MC, University Medical Center Rotterdam, Department of Microbiology and Infectious Diseases, Rotterdam, The Netherlands; 3 Mycetoma Research Centre, University of Khartoum, Khartoum, Sudan; 4 Institut Pasteur, Molecular Mycology Unit, National Reference Center for Invasive Mycoses and Antifungals, CNRS UMR2000, Paris, France; 5 Center of Expertise in Mycology of Radboudumc / Canisius Wilhelmina Hospital, Nijmegen, The Netherlands; 6 Institute for Biodiversity and Ecosystem Dynamics, University of Amsterdam, Amsterdam, The Netherlands; 7 Faculty of Medical Laboratory Sciences, University of Khartoum, Khartoum, Sudan; Wellcome Trust Sanger Institute, UNITED KINGDOM

## Abstract

The genus *Madurella* comprising four species, *M*. *fahalii*, *M*. *mycetomatis*, *M*. *pseudomycetomatis*, and *M*. *tropicana*, represents the prevalent cause of eumycetoma worldwide. The four species are phenotypically similar and cause an invariable clinical picture, but differ markedly in their susceptibility to antifungal drugs, and epidemiological pattern. Therefore, specific identification is required for optimal management of *Madurella* infection and to reveal proper epidemiology of the species. In this study, a novel multiplex real-time PCR targeting the four *Madurella* species was developed and standardized. Evaluation of the assay using reference strains of the target and non-target species resulted in 100% specificity, high analytical reproducibility (R2 values >0.99) and a lowest detection limit of 3 pg target DNA. The accuracy of the real-time PCR was further assessed using biopsies from eumycetoma suspected patients. Unlike culture and DNA sequencing as gold standard diagnostic methods, the real-time PCR yielded accurate diagnosis with specific identification of the causative species in three hours compared to one or two weeks required for culture. The novel method reduces turnaround time as well as labor intensity and high costs associated with current reference methods.

## Author summary

Mycetoma, a progressive and disfiguring disease, is one of the neglected tropical diseases, caused by both bacteria and fungi. Eumycetoma is the fungal type and mainly caused by species of the genus *Madurella*. *Madurella mycetomatis* is the most prevalent species worldwide. However, other species such as *M*. *fahalii*, *M*. *pseudomycetomatis*, and *M*. *tropicana* can also cause mycetoma and have a different susceptibility towards the drug used for treating mycetoma patients. Currently, we lack a rapid and non-culture-based technique that can readily identify these four species from clinical samples. Due to its sensitivity, and specificity, real-time PCR is re-recognized by European Organization for Research and Treatment of Cancer (EORTC) to directly identify fungal agents from clinical samples. We developed and validated a multiplex real-time PCR-based technique using the least expensive chemistry to identify *Madurella* species within 3–4 hours. Development of such a technique will allow rapid diagnosis of eumycetoma and timely initiation of appropriate antifungal therapy.

## Introduction

Mycetoma, a neglected tropical disease of the subcutaneous tissue, is characterized by progressive tumefaction and formation of grains and multiple draining sinuses, leading to massive soft tissue destruction and disfigurement [1, 2]. The chronic and disabling nature of mycetoma carries a significant economic and social burden to the inhabitants of the endemic areas in the tropics [2, 3]. In the presence of classical clinical features, *i*. *e*. sinuses in the skin and grain discharge, mycetoma can easily be recognized from other skin diseases, while proper diagnosis with identification of the etiologic agent requires a combination of diagnostic tools. Imaging, cytology, and histology are used to confirm the clinical diagnosis and to establish the extent and the nature of the agent, which is either a bacterium (actinomycetoma) or a fungus (eumycetoma) [4, 5]. However, due to the insufficient specificity, these methods do not allow conclusive diagnosis for which accurate identification of the causative pathogen is required [4].

Although, historically a limited number of species was reported to cause eumycetoma, the advent of molecular approaches enabled recognition of a plethora of novel agents [6]. *Madurella* as the most prevalent causative pathogen remains responsible for 70% of the eumycetoma cases in arid climate regions of Northeastern Africa and India [3, 7]. *Madurella* contains four species, *viz*. *M*. *mycetomatis*, which was described in 1905 and is the type species of the genus, and *M*. *fahalii*, *M*. *pseudomycetomatis*, and *M*. *tropicana* which were recognized after 2010 [8, 9]. Except for *M*. *mycetomatis* the commonly reported species worldwide, the epidemiology of the other *Madurella* remains as yet unknown. *Madurella fahalii* was described in 2012 from a eumycetoma patient in Sudan, a country that is highly endemic for mycetoma [8]. In Sudan, cases that show presence of black grains in histology or fine needle aspiration cytology are usually diagnosed as *M*. *mycetomatis* eumycetoma [6], but *M*. *fahalii* also causes infection with black grains. Since *M*. *fahalii* is not inhibited by itraconazole *in vitro*, it is crucial to be able to perform proper species identification [8]. Furthermore, occurrence of marked differences in antifungal susceptibility between *Madurella* species and other agents of black grain eumycetoma necessitate the development and implementation of a species-specific identification system [7, 10]. Correct identification will assist in the administration of appropriate antifungal therapy, and will help to elucidate the epidemiology and distribution of agents of eumycetoma.

Due to the lack of sporulation and other *in vitro* phenotypic characteristics in *Madurella* species, morphological assays have limited diagnostic value [4]. Alternatively, sequencing of ribosomal internal transcribed spacer (ITS) as the gold standard technique for species identification, and protein-based approaches such as Matrix-Assisted Laser Desorption Ionization-Time-Of-Flight Mass Spectrometry (MALDI-TOF MS) are regarded as highly reliable identification tools [4, 11]. However, long turnaround time, high costs and the requirement for skilled laboratory staff interfere with establishment of sequencing and MALDI-TOF MS in developing countries. Rapid and sensitive isothermal amplification assays such as loop-mediated isothermal amplification (LAMP), rolling-circle amplification (RCA), and recombinase polymerase amplification (RPA) eliminated the need for culture and PCR machinery, but they lack the multiplexing capability [12, 13]. An appealing alternative can then be a real-time PCR assay.

Real-time PCR has become increasingly utilized in clinical diagnostics as one of the preferred assays for rapid diagnosis of fungal infection. The high sensitivity and the capability of detecting non-viable organisms are the significant advantages of these assays [14]. Besides, the detection of multiple targets in a single tube simultaneously has placed Real-time PCR on top of the diagnostic arsenal [14]. Herein, we developed a sensitive and specific SYBR-Green I-based real-time PCR assay that can specifically identify *M*. *fahalii*, *M*. *mycetomatis*, *M*. *pseudomycetomatis*, and *M*. *tropicana*. We also developed a sensitive and specific duplex qPCR assay for *M*. *mycetomatis* and *M*. *fahalii* and single-plex qPCR assay for *M*. *mycetomatis*. These assays allow reliable and rapid diagnosis of the main agents of eumycetoma and can be applied in clinical settings.

## Methods

### Ethics approval

This study was reviewed and approved by the Ethical Review Board of Soba University Hospital Ethical Committee (Khartoum, Sudan). Written informed consents were obtained from all patients and their data were processed anonymously to ensure confidentiality.

### Strains

Strains used in this study were acquired from the CBS-KNAW reference collection of the Westerdijk Fungal Biodiversity Institute, The Netherlands; the Mycetoma Research Centre (MRC), Sudan; the Pasteur Collection of Fungi (UMIP), France; and the National Reference Center for Invasive Mycoses and Antifungals (NRCMA), France. In total 47 strains were included in this study (Table 1), of which 26 belonged to the genus *Madurella*, 9 to other members of the family *Chaetomiaceae*, 9 to black-grain mycetoma species in the order *Pleosporales*, 2 to *Aspergillus* and 1 *Rhizopus* species. Lyophilized, cryo-preserved or fresh mycelial material from the strains was inoculated onto Malt Yeast Extract agar (MEA, Oxoid, UK) plates and incubated for 2 to 3 weeks at temperature ranging 24–30°C.

**Table 1:** Strains used as the blind test set for the evaluation of analytical sensitivity and specificity of *Madurella* real-time PCR assay *.

	Isolate Number	Species Name	Species Affiliation
1	CBS 332.67	*Achaetomium globosum*	*Sordariales*, *Chaetomiaceae*
2	CBS 122.55	*Aspergillus niger*	*Eurotiales*, *Aspergillaceae*
3	CBS 139335	*Aspergillus flavus*	
4	CBS 487.48	*Berkeleyomyces basicola*	*Sordariales*, *Chaetomiaceae*
5	CBS 731.71	*Chaetomium homopilatum*	
6	CBS 178.84	*Chaetomium murorum*	
7	CBS 813.73	*Chaetomium succineum*	
8	CBS 414.73	*Chaetomium variosporum*	
9	CBS 144164	*Collariella causiiformis*	
10	CBS 132257	*Falciformispora senegalensis*	*Pleosporales*, *Trematosphaeriaceae*
11	CBS 132272	*Falciformispora senegalensis*	
12	CBS 196.79	*Falciformispora senegalensis*	
13	CBS 200.79	*Falciformispora tompkinsii*	
14	CBS 201.79	*Falciformispora tompkinsii*	
15	CBS 129176	*Madurella fahalii*	*Sordariales*, *Chaetomiaceae*
16	CBS 102793	*Madurella fahalii*	
17	UMIP595.60	*Madurella fahalii*	
18	CNRMA9.616	*Madurella fahalii*	
19	CBS 132259	*Madurella mycetomatis*	
20	CBS 132262	*Madurella mycetomatis*	
21	CBS 132588	*Madurella mycetomatis*	
22	CBS 132419	*Madurella mycetomatis*	
23	CBS 132297	*Madurella mycetomatis*	
24	CBS 110087	*Madurella mycetomatis*	
25	CBS 132267	*Madurella mycetomatis*	
26	CBS 132263	*Madurella mycetomatis*	
27	CBS 132270	*Madurella mycetomatis*	
28	CBS 132285	*Madurella mycetomatis*	
29	CBS 110359	*Madurella mycetomatis*	
30	CBS 109801	*Madurella mycetomatis*	
31	CBS 132266	*Madurella mycetomatis*	
32	CBS 248.48	*Madurella pseudomycetomatis*	
33	CBS 102791	*Madurella pseudomycetomatis*	
34	CBS 216.29	*Madurella pseudomycetomatis*	
35	CBS 129177	*Madurella pseudomycetomatis*	
36	CBS 217.55	*Madurella pseudomycetomatis*	
37	CBS 331.50	*Madurella tropicana*	
38	CBS 219.92	*Madurella tropicana*	
39	CBS 201.38	*Madurella tropicana*	
40	CBS 206.47	*Madurella tropicana*	
41	CBS 252.60	*Medicopsis romeroi*	*Pleosporales*, *Neohendersoniaceae*
42	CBS 132878	*Medicopsis romeroi*	
43	CBS 391.34	*Rhizopus delemar*	*Mucorales*, *Rhizopodaceae*
44	CBS 160.80	*Thielavia subthermophila*	*Sordariales*, *Chaetomiaceae*
45	CBS 510.74	*Thielavia subthermophila*	
46	CBS 332.50	*Trematosphaeria grisea*	*Pleosporales*, *Trematosphaeriaceae*
47	CBS 246.66	*Trematosphaeria grisea*	

*CBS, Centraalbureau voor Schimmelcultures; UMIP, Institute Pasteur Collection of Fungi; CNRMA, National Reference Center for Invasive Mycoses and Antifungals.

### Clinical samples

Thirteen clinical samples obtained from patients seen at the MRC, Khartoum, Sudan were included in the study. The deep surgical biopsies were collected for diagnostic purposes; 11 biopsies showed black grain in the histological sections, while in two biopsies no grains were observed and therefore, we used them as negative controls. Both positive and negative biopsies were cultured on Sabouraud’s Glucose Agar (SGA) and the identity of the isolates was established by ITS sequencing. These clinical samples were retrospectively collected and in a context of a blind test set they were assigned with numerical code numbers (1–13). The assessor of the qPCR assay was not aware of the identity of DNA samples (for both DNA samples derived from pure cultures and those obtained from clinical samples).

### DNA extraction

Strains were grown on 2% MEA or SGA plates and incubated for 2 weeks. Mycelia were harvested and DNA was extracted using cetyltrimethyl ammonium bromide (CTAB) and glass beads method as described previously by Moller *et*. *al*. [15]. The strains were then identified to species level by amplification and sequencing of the rDNA ITS and part of β-tubulin gene regions (S1 Fig).

Patient’s biopsies were maintained at −20°C in sterile physiological saline and the DNA was isolated using DNeasy Plant Mini Kit (Qiagen, Hilden, Germany) after bead-beating with 2 mm metal beads as described by Ahmed et al. [13].

Purity of extracted DNA was assessed using NanoDrop ND-1000 spectrophotometer (Thermo Fisher Scientific, Wilmington, USA) and on 1% agarose gels. In order to accurately measure the DNA concentration, samples were subjected to QuBit Broad Range kit (Thermo Fisher) that specifically binds and measures DNA molecules. Genome sizes of target species were calculated with the assumption that one ng DNA of *C*. *albicans* was equal to 100,000 genomes [16]. The size of the *M*. *mycetomatis* genome (36.7 Mbps) is approximately three times of that of *C*. *albicans* (14.28 Mbps). As a result, three ng of DNA of target species were considered to represent 100,000 genomes [17]. As the genome size of the remaining target species is unknown, the genome size as *M*. *mycetomatis* was considered for their genome number calculations.

### Primer design

Sequences of ITS and β-tubulin from target and non-target species were retrieved from NCBI (www.ncbi.nlm.nih.gov) and CBS-KNAW databases (http://www.westerdijkinstitute.nl) and combined with the sequences obtained in this study. Alignment and primer design used the Geneious software (Biomatters ApS, Katrinebjerg, Denmark). Primers were placed in the most stable positions with the least and the highest degree of variation with target species and non-target species, respectively. Various features of primers including delta G and Tm values were evaluated by the online Oligo Analyzer software (eu.idtdna.com). UMelt software (https://www.dna.utah.edu/umelt/um.php) was used to assess the amplicon melt peaks generated by each primer pair. Primers meeting the following criteria were selected for specificity and sensitivity testing: a) Lack of cross-reaction with other target and non-target species, b) Minimum self and heterodimers, c) Melting temperature (Tm) values of 60–62°C, d) Generating amplicons with distinctive and single melt peaks in multiplex qPCR reactions, and e) Exhibiting satisfactory efficiency and R^2^ values. Primers that failed to amplify the DNA of target species or those showing Tm overlap with the other target and non-target species were excluded from this study (Table 2). Primers were manufactured by Integrated DNA Technology Company (IDT, Leuven, Belgium).

**Table 2:** List of designed primers in this study. Some primers due to cross-reaction, Tm overlap with the other species, and lack of enough efficiency and R^2^ values were excluded. Primers with distinctive Tm values, reasonable efficiency and R^2^ values were selected for further experiments.

Successful primers	Primer names	Primer sequences	Target species	Loci
	Myc-F3 Myc-R3 (PF3/R3-Myc)	CTCCCGGTAGTGTAGTGT* CAGAAGACTCAGAGAGGCC	*M*. *mycetomatis*	ITS
	Fah-F1 Fah-R1 (PF/R-fahalii)	CATTGTGAACCTACCCAAAA CATACAAAGTACAGGGTTTATGTA	*M*. *fahalii*	ITS
	Myc-F1 Myc-R1	GTTCGATGGCCTCCGCTG* TTGCCCTGGAAAGGCCCTC	*M*. *mycetomatis*	ß-tubulin
	Myc-F2 Myc-R2 (PF2/R2-specific-Myc)	TGACCGTCGGCGTCTCTT TAGGCTGTCAGAAAACACATCG	*M*. *mycetomatis*	ß-tubulin
	MPT-F MPT-R (PF/R-Universal)	GTGTCGGGAACTGACGAGG CCTTGCTGGCCCTTTGC	*M*. *pseudomycetomatis*, *M*. *tropicana* *M*. *mycetomatis*	ß-tubulin
Failed primers	Mad-UniF Mad-UniR	CCATTGTGAACCTACCCAA CAGAGACTCAGAGAGGCC	*M*. *pseudomycetomatis*, *M*. *tropicana* *M*. *mycetomatis* *M*. *fahalii*	ITS
	MPT-F1 MPT-R1	TTGTGAACCTACCCAAAAAA AGCAAACAGGGTGTTGTATAAT	*M*. *pseudomycetomatis*, *M*. *tropicana* *M*. *mycetomatis*	ITS
	MPT-F Myc-R0 Pse-R2 Tropi/fah-R2	CCCCGAGCGTAGTAGTTA GGGGTAAAAATGAGTTGGGC AACCTTGGGGGGGTAAT AACCTTGGGGTAAWGGGT	*M*. *pseudomycetomatis*, *M*. *tropicana* *M*. *mycetomatis* *M*. *fahalii*	ITS

### qPCR conditions

PCR reaction was set up to a final volume of 20 μl and contained the following ingredients: 10μl PowerUp SYBR Green master mix (A25742 Thermo Fisher), 5 pMol primers (PF3/R3-Myc and PF/R-Fahalii for a duplex qPCR reaction, PF/R-universal and PF/R-Fahalii for tetraplex qPCR reaction, and PF/R-specific Myc for *M*. *mycetomatis*-specific qPCR reaction), 3 ng of DNA (1 μl), and MilliQ water to adjust the volume to 20 μl.

PCR reactions were performed using an ABI 7500 fast PCR device (Thermo Fisher) and the following PCR program was used: one cycle of 50°C for 2 min and 95°C for 3 min, followed by 40 cycles of 95°C for 15 sec and 60°C for 30 sec. Upon finishing the PCR program, PCR products were subjected to a melt curve analysis program that used an increment of 0.5°C/sec starting at 60°C and terminated at 95°C. The data obtained from qPCR were analyzed by ABI 7500 Software V2.3 (Thermo Fisher).

### Specificity and sensitivity

In order to measure the sensitivity of qPCR assays, serial dilutions were prepared of DNA samples of the type strain; *M*. *fahalii* (CBS 129176), *M*. *mycetomatis* (CBS 109801), *M*. *pseudomycetomatis* (CBS 129177), and *M*. *tropicana* (CBS 201.38), with a log10 over four orders of magnitude starting at 3 ng and ending at 3 pg genomes. This experiment was performed in duplicate and on three consecutive days.

To test specificity, a blinded test set containing 47 isolates (*M*. *fahalii*, *n =* 4, *M*. *mycetomatis*, *n* = 13, *M*. *pseudomycetomatis*, *n* = 5, *M*. *tropicana*, *n* = 4, and 21 non-target species) was subjected to the duplex, tetraplex, and *M*. *mycetomatis*-specific (MM-specific) qPCR assays (Table 1).

Primers passing both sensitivity and specificity experiments were selected for the next step of testing DNA samples obtained from patients (Tables 2 and 3). Cycle thresholds were adjusted in such a way that they could differentiate target from non-target species.

**Table 3:** Primers designed in this study and lead to successful identification of target species*.

Primer name	Primer sequence	Target species	Melting temperature	PCR product size	LOD (Genome)	Efficiency (%)	R^2^ values
PF/R-Universal	GTGTCGGGAACTGACGAGG CCTTGCTGGCCCTTTGC	*M*. *mycetomatis* *M*. *pseudomycetomatis* *M*. *tropicana*	84.17±0.19°C 83.07±0.13°C 83.6±0.09°C	95 bps 95 bps 95 bps	100 100 100	99.5% 94.18% 93.4%	0.991 0.996 0.997
PF/R-fahalii	CATTGTGAACCTACCCAAAA CATACAAAGTACAGGGTTTATGTA	*M*. *fahalii*	89.13±0.41°C	116 bps	100	70%	0.983
PF2/R2-specific-Myc	TGACCGTCGGCGTCTCTT TAGGCTGTCAGAAAACACATCG	*M*. *mycetomatis*	84.91±0.18°C	166 bps	100	96.5%	0.997
PF3/R3-Myc	CTCCCGGTAGTGTAGTGT CAGAAGACTCAGAGAGGCC	*M*. *mycetomatis*	84.1±0.19°C	99 bps	100	90%	0.99

*LOD, Limit of Detection.

### Evaluation of performance of qPCR assays with patients’ DNA samples

DNA samples of 13 patient biopsies were prepared in a blinded manner and used to assess the performance of our qPCR assays. One microliter of those DNA samples was subjected to duplex, tetraplex, and *M*. *mycetomatis*-specific qPCR reactions.

### Statistical analysis

Statistical analysis and graph preparations were performed by GraphPad Prism software (La Jolla, California, USA). Concordance between the qPCR and the gold standard method (culture and sequencing) was evaluated using kappa test and expressed as *K* value.

## Results

### Standardization of the qPCR assay

**Fig 1:**
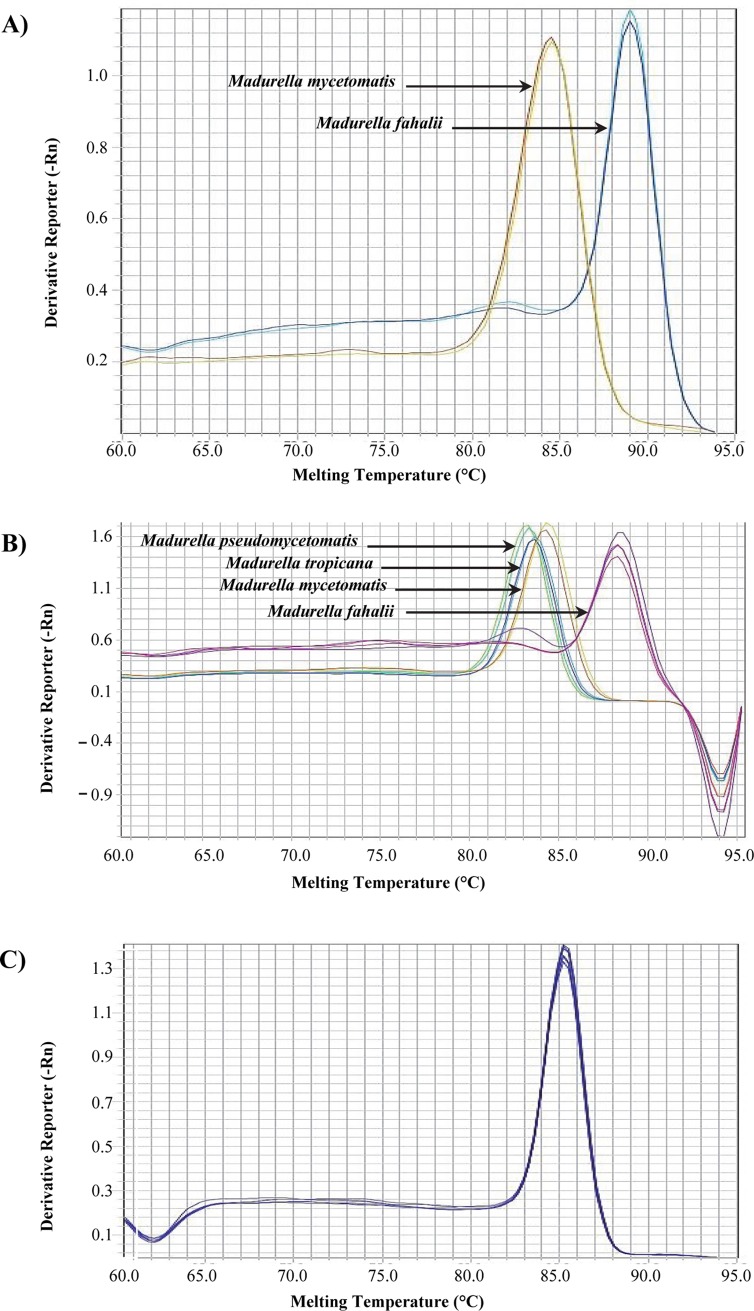
Identification of *Madurella* species using qPCR assay. (A) Identification of *M*. *fahalii* and *M*. *mycetomatis* using duplex qPCR assay. (B) Identification of all target species using a tetraplex qPCR assay. (C) Identification of *M*. *mycetomatis* using a *M*. *mycetomatis*-specific qPCR assay.

Initially, the ITS sequence was utilized as a target locus for designing species-specific and universal primers. Only PF3/R3-Myc targeting *M*. *mycetomatis* (84.1±0.18°C) and PF-R-Fahalii targeting *M*. *fahalii* (89.13±0.41°C) resulted in successful specific and distinguishable melt curves (Tables 2 and 3 and Fig 1A). These two primers were then used to optimize duplex qPCR that can specifically detect *M*. *mycetomatis* and *M*. *fahalii*. Since universal primers based on ITS failed in specific identification of the four target species, the β-tubulin gene was used for the same purpose. A universal primer pair derived from β-tubulin successfully resulted in distinctive melting temperatures of 83.07±0.13°C, 83.6±0.09°C, and 84.17±0.19°C and allowing distinction of three target species, *viz*. *M*. *pseudomycetomatis*, *M*. *tropicana*, and *M*. *mycetomatis*, respectively. *Madurella fahalii* could not be distinguished with this set of primers. In order to make a comprehensive multiplex qPCR that can unambiguously identify all four species, primer set PF/R-Fahalii derived from ITS was added to the β-tubulin universal primers in the same reaction (Fig 1B, Tables 2 and 3). Using this combination, four distinct melting peaks corresponding to the four target species were revealed.

In order to develop a specific qPCR for *M*. *mycetomatis*, two more primer pairs based on β-tubulin with an increased difference of melting temperature from the closest species in the multiplex PCR were designed. These primers generated amplicons with Tm values of 84.5±0.1°C (Myc-F1/R1) and 84.9±0.18°C (Myc-F2/R2) (Fig 1C, Tables 2 and 3). Despite successful amplification of *M*. *mycetomatis* using both primers, MycF2/R2 was used for the *M*. *mycetomatis*-specific qPCR as it showed a higher and more distinguishable Tm value from *M*. *pseudomycetomatis* and *M*. *tropicana*. In general, primers that were designed based on β-tubulin showed a higher rate of success than those based on the ITS region, which might be due to a higher GC content of the target region of the latter.

In the next step, in view of sensitivity testing all three qPCR reactions were subjected to serially diluted DNA samples (3 ng–3 pg) of target species. Sensitivity testing revealed that *M*. *mycetomatis*, *M*. *pseudomycetomatis*, and *M*. *tropicana* were successfully and efficiently amplified with an efficiency of ≥90% and the R^2^ values >0.99; for *M*. *fahalii* the efficiency was 70%. Moreover, 3 pg DNA of all target species were successfully detected and distinguished by the three qPCR assays.

### A high degree of specificity was confirmed by blinded DNA samples obtained from cultures

**Fig 2:**
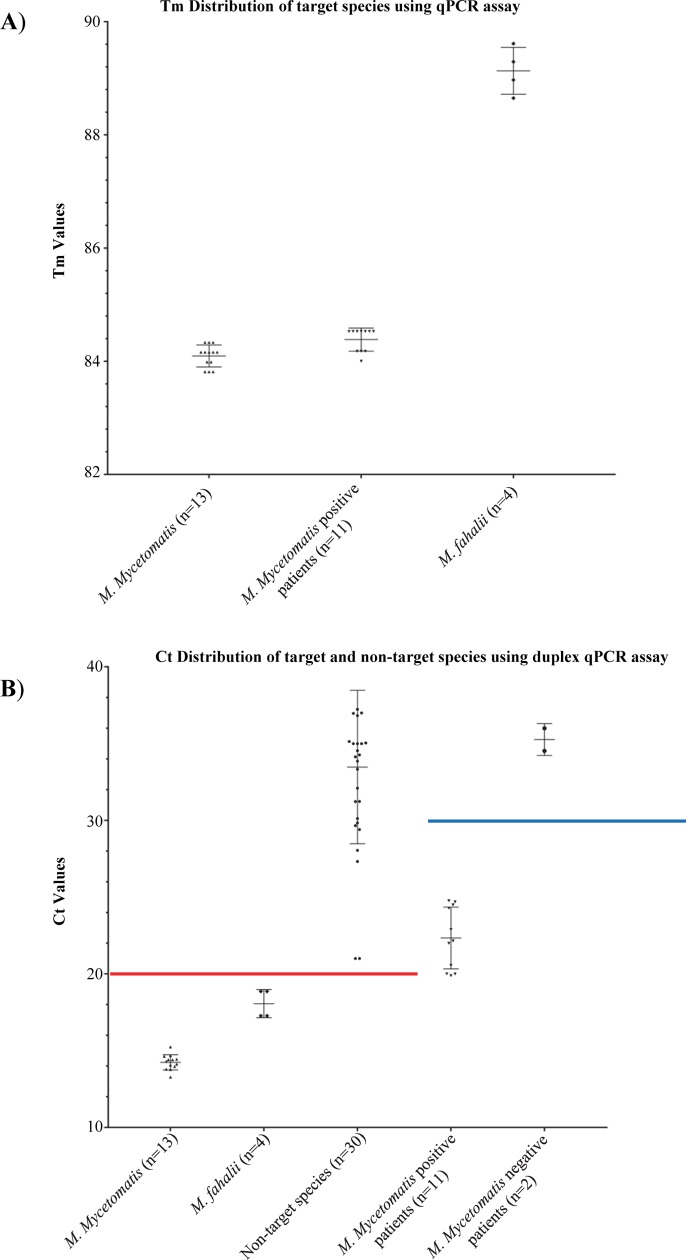
Specificity of duplex qPCR assay of *M*. *fahalii* and *M*. *mycetomatis*. (A) Tm values obtained from duplex qPCR assay for *M*. *fahalii* and *M*. *mycetomatis*. (B) Duplex qPCR assay with a blinded test set and DNA samples obtained from patients yielded 100% specificity and sensitivity if the threshold cycles were set at<20 (red line), and <30 (blue line), respectively.

**Fig 3:**
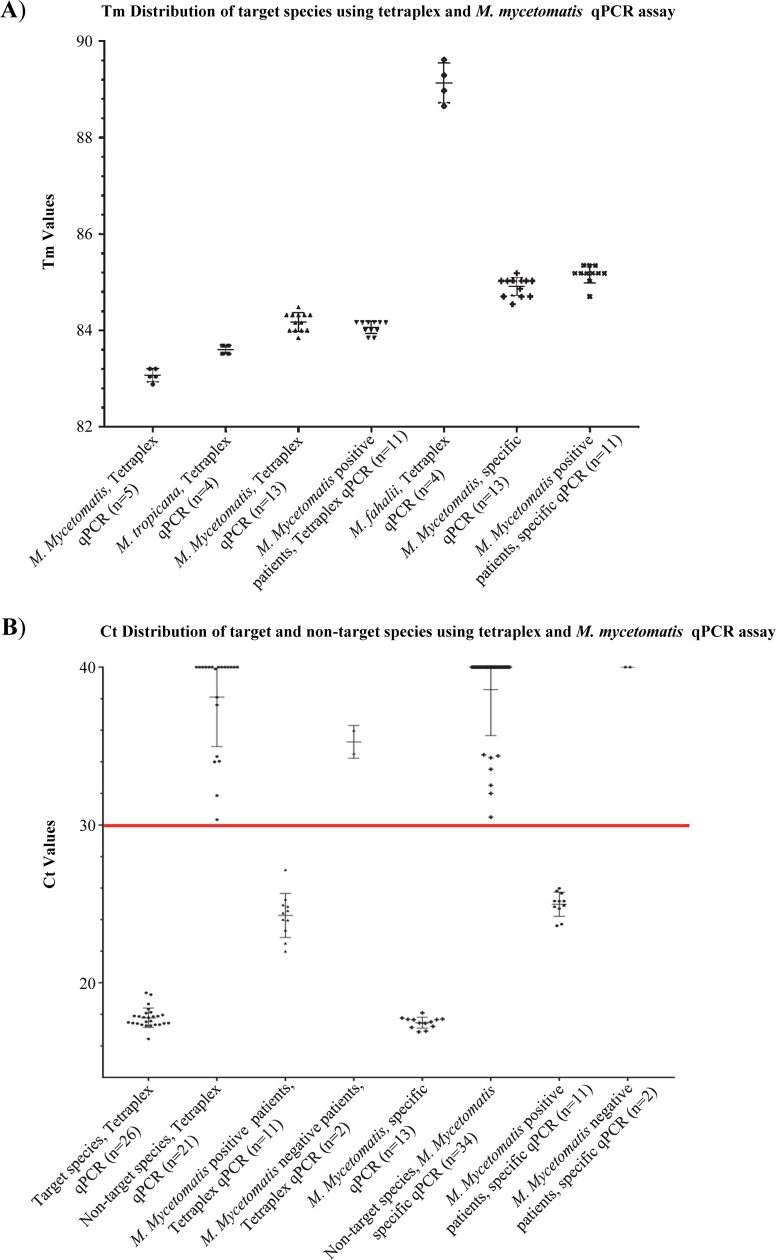
Specificity and sensitivity of singleplex and tetraplex qPCR assays of *Madurella* species. (A) Tm values obtained from tetraplex and *M*. *mycetomatis*-specific qPCR assays for *M*. *fahalii*, *M*. *mycetomatis*, *M*. *pseudomycetomatis*, *and M*. *tropicana*. (B) Tetraplex and MM-specific qPCR with a blinded test set and DNA samples obtained from patients yielded 100% specificity and sensitivity if the threshold cycles <30 (red line).

**Fig 4:**
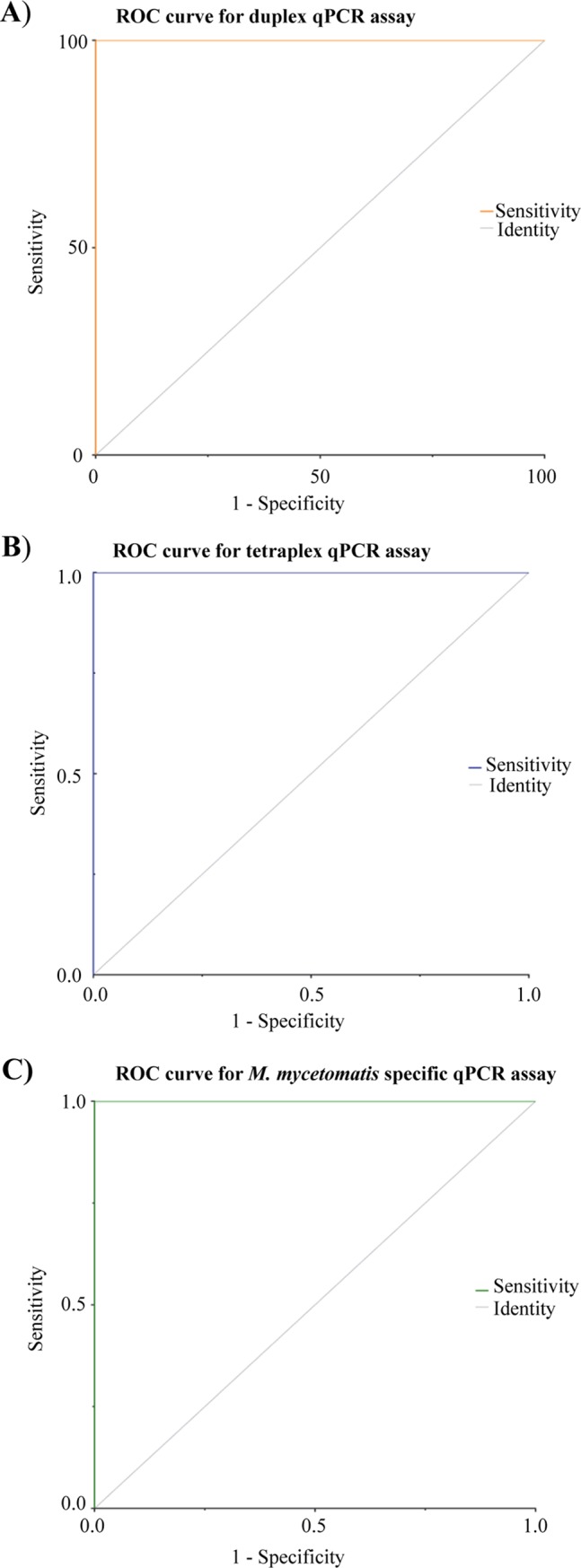
Receiver operation characteristic curve (ROC) obtained for the three qPCR assays revealed 100% specificity when subjected to blinded test sets.

In the third phase, successful qPCR reactions were challenged by a blinded test set that contained 26 target and 21 non-target species isolates. Our duplex qPCR assay resulted in 100% specificity when the threshold for *M*. *mycetomatis* and *M*. *fahalii* were set at Ct value of <20 and <38, respectively (Figs 2 and S2). Some of the non-target species yielded melting curves similar to that of *M*. *fahalii* with Ct values>38, therefore Ct values <38 and >38 were considered as positive and negative for this species, respectively. Ct values of <30 yielded 100% specificity for the tetraplex and *M*. *mycetomatis*-specific qPCR assays (Fig 3). As the primer sets Pf/R-Fahalii were used in both duplex and tetraplex qPCR assays, the same Ct value (<38) was considered for both qPCR reactions. Receiver operation characteristics curves (ROC) showed that with the established threshold cycle values, 100% specificity was obtained for the three qPCR assays (Fig 4).

### A high degree of sensitivity and specificity was confirmed by blind DNA samples obtained from patients

Following success in sensitivity and specificity testing, accepted primers were evaluated by 13 DNA samples obtained from documented *M*. *mycetomatis*-positive and negative patients. In accordance with histology, culture and ITS sequencing, both 11 positive and the 2 negative patients were successfully identified by our qPCR assays within 3 hours starting from DNA isolation (Figs 2, 3 and S2). The kappa test revealed a *K* value of 1.00 (95% CI 1.00 to 1.00) which indicates perfect agreement. The diagnostic sensitivity and specificity of the qPCR assays were 100% (95% CI, 71.51% to 100%) and 100% (95% CI, 15.81% to 100%), respectively.

## Discussion

In the present study, we developed the first multiplex real-time PCR assay that is able to detect and identify the four currently accepted *Madurella* species, *viz*. *M*. *fahalii*, *M*. *mycetomatis*, *M*. *pseudomycetomatis*, and *M*. *tropicana*. As the real-time PCR is one of the fastest methods available for diagnosis of fungal infections [18], we herewith provide a rapid, sensitive, and specific diagnostic assay for eumycetoma caused by *Madurella* species. Currently, the only available tool to ascertain the species identity in *Madurella* is by application of sequencing rDNA ITS and protein-coding genes such as ß-tubulin and RNA polymerase II subunit [8, 19]. Since these markers showed optimal performance in previous studies, we designed our universal and species-specific primers based on the ITS and the ß-tubulin gene sequences of the target species [12, 13, 20]. The utility of the ITS for species-specific PCR identification of *Madurella* has previously been described by Ahmed *et al*. [20], who designed the first species-specific primers for identification of *M*. *mycetomatis*. In addition, specific LAMP and RPA primers have also been developed for the same species using the ITS region [13]. Despite the good discriminatory power of ITS, our universal primers based on this region could not sufficiently identify the four target species. Therefore, as a first step, a set of primers specific for detection of only *M*. *mycetomatis* and *M*. *fahalii* and based on the ITS region were designed. This primer set can be used in areas where these two species are preponderant, such as in Sudan, which is one of the hyperendemic regions with mycetoma, and in 70% of the cases the causative agent has been conventionally identified as *M*. *mycetomatis* [4, 21].

In order to develop a single assay that can be used to detect all four *Madurella* species, we designed another set of universal and species-specific primers based on the ß-tubulin region. When this set was combined with *M*. *fahalii* specific ITS primers, an optimal result was obtained. Our multiplex qPCR assay yielded 100% specificity with a blind test set containing the most closely- and distantly-related species to *Madurella* [22]. To further evaluate the diagnostic performance and prove that the assay is capable of identifying the target species in clinical specimens, 13 blinded DNA samples derived from *M*. *mycetomatis*-positive (*n* = 11) and negative patients (*n* = 2) were subjected to our qPCR assay. The result showed 100% diagnostic sensitivity and specificity, as successful differentiation of positive and negative patients and specific identification of the causative agent was achieved.

Although culture is regarded as the gold standard in the field of medical mycology, this technique greatly suffers from lack of specificity and is time-consuming [23]. Apart from these factors, in order to obtain positive results for *Madurella* agents, culture requires viable grains, while our qPCR due to exhibiting a high degree of sensitivity and specificity can be applied on discharge samples that contain non-viable grains, hence, it may not require invasive procedures for obtaining sterile samples [4,24].

Despite the wide use in the field of diagnostics, as yet, no real-time PCR assay has been developed for diagnosis of eumycetoma agents. The technique has mainly been used for systemic fungal pathogens like *Candida* and *Aspergillus* [25]. Lu *et al*. [26] and Castelli *et al*. [27] developed real-time PCR for detection of *Scedosporium* species, a fungus which is frequently found as a colonizer in the lung of cystic fibrosis patient but also can cause white grain eumycetoma in rare cases [28]. The real-time PCR developed by Lu *et al*. was based on ß-tubulin as a target for species identification [26]. However, using this marker, the authors were unable to distinguish the six species concerned, which could be due to the uncertain taxonomic status of strains of respective species in this genus, as nowadays *S*. *apiospermum* and *S*. *boydii* are referred to as ‘*S*. *apiospermum* species complex’ [28].

Elhassan *et al*. [29] evaluated the use of real-time PCR for identification of clinical isolates from actinomycetoma patients using *strb-1* as a target gene. The study represents the first application of real-time PCR for diagnosis of actinomycetoma in an endemic region. *Streptomyces* species were identified in 7 samples from patients originating from Sudan [29].

Only recently, culture-free diagnostic tests have been introduced for black grain eumycetoma diagnosis [13,30]. This was previously hampered by the great difficulty in obtaining pure DNA from the compact grains. Ahmed *et al*. [13] described an efficient DNA isolation method using bead beating with metal beads. Combining this DNA isolation method with our real-time PCR assay resulted in a rapid and specific diagnosis of target species. Despite the fact that other PCR/isothermal-based technologies have been used for direct detection of *Madurella* as well, these are all single-plex assays [13]. Furthermore, the species-specific PCR-RFLP technique developed by Ahmed *et al*. for *M*. *mycetomatis* detection requires application of specific restriction enzymes which is time-consuming, laborious, costly, and even opening tubes increases the risk of cross contamination [20]. In contrast, our qPCR is multiplex and performs the assay in a gel-independent and closed-tube manner, and as a result, it minimizes the chance of cross-contamination and false-positive results.

Fraser *et al*. [11] in 2017 developed a MALDI-TOF MS library that successfully identified 13 *M*. *mycetomatis* and 2 *M*. *fahalii*. This method, however, similar to Sanger sequencing, requires a costly purchase and maintenance that prevents their implementation in low-resource countries. Moreover, they are culture-dependent and thus time-consuming. Although the high capital equipment costs and the requirement for well-trained laboratory staff might also limit the use of real-time PCR in resource-limited regions endemic with mycetoma, nevertheless it is still simpler and faster than culture and DNA sequencing or MALDI-TOF. The affordability, reproducibility, and construction of home-made robust PCR machines using the most basic off-the-shelf appliance are the appealing features that will allow this machinery to find its niches even in developing countries. In our qPCR we used SYBR-Green I which is currently the cheapest option for such a technique.

In conclusion, we have developed a rapid and accurate diagnostic assay that is able to simultaneously identify *M*. *fahalii*, *M*. *mycetomatis*, *M*. *pseudomycetomatis*, and *M*. *tropicana*. The assay can be used in both culture-dependent and -independent manners. The study had some limitations, including that our qPCR assays were not evaluated with the clinical samples infected with other *Madurella* agents due to the unavailability of such material. Therefore, screening a larger number of patient biopsies and direct discharges from different endemic regions are warranted to reveal the real performance of the assay. Despite those limitations, the high sensitivity, specificity and short turnaround time represent a significant advantage of this method.

## Supporting information

S1 Fig Amplification of ITS region of Madurella species. PCR amplification of ITS region of the four *Madurella* species using primers V9G and LS266. Lane M, DNA ladder; lane 1and 2, *M*. *fahalii*; lane 3 and 4, *M*. *tropicana*; lane 5 and 6, *M*. *pseudomycetomatis*; Lane 7–10 *M*. *mycetomatis*; Lane 11, negative control.
(TIF)

S2 Fig Standards for Reporting of Diagnostic Accuracy (STARD) flow chart of Madurella real-time PCR. (TIF)
