# Supplementary figures and images for: Madurella real-time PCR, a novel approach for eumycetoma diagnosis
Source: PLoS Negl Trop Dis. 2020 Jan 15;14(1):e0007845. doi: 10.1371/journal.pntd.0007845 (PMC6986762; doi:10.1371/journal.pntd.0007845)

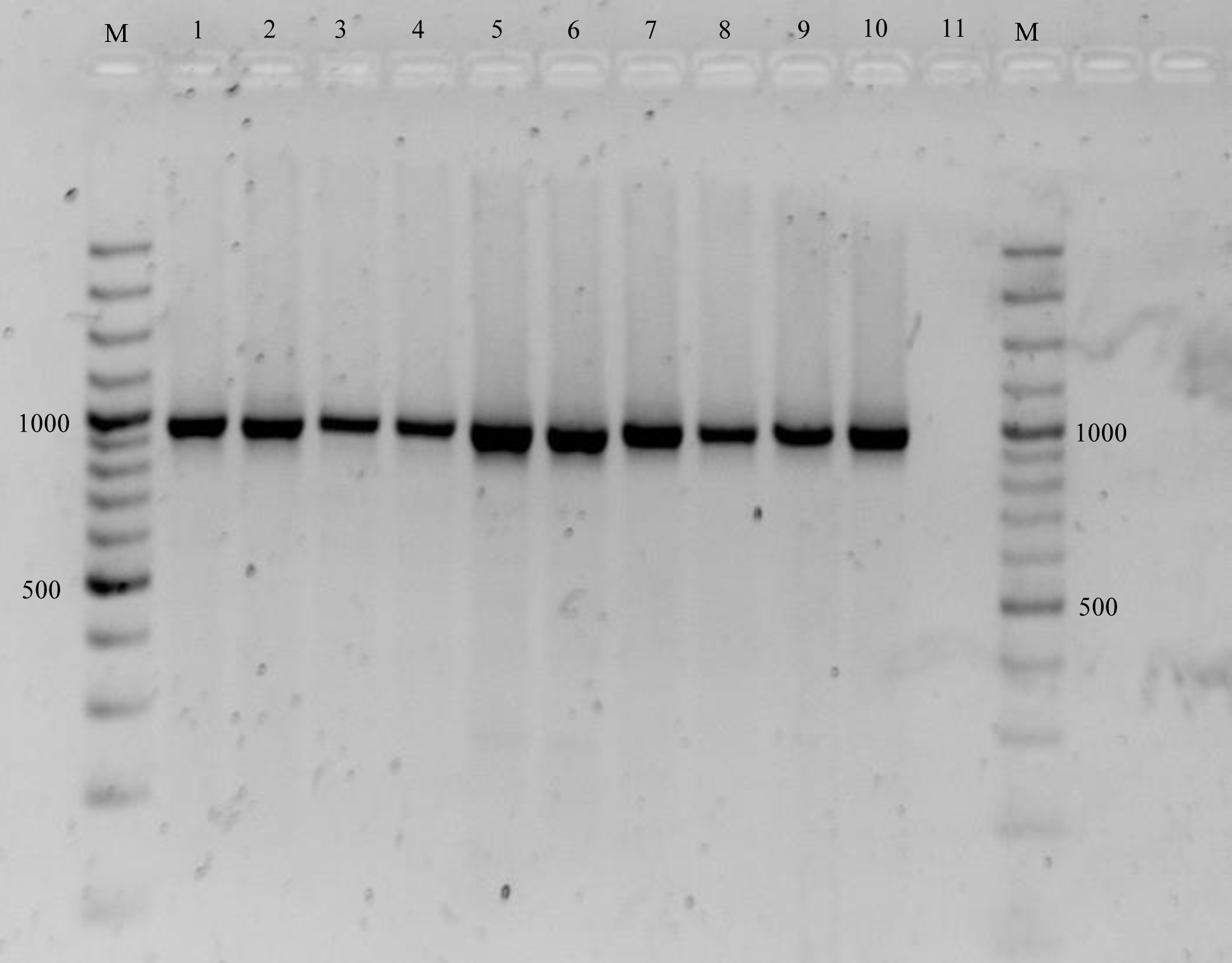

Supplement: S1 Fig — PCR amplification of ITS region of the four Madurella species using primers V9G and LS266. Lane M, DNA ladder; lane 1and 2, M. fahalii; lane 3 and 4, M. tropicana; lane 5 and 6, M. pseudomycetomatis; Lane 7–10 M. mycetomatis; Lane 11, negative control. (TIF) [file pntd.0007845.s001.tif]

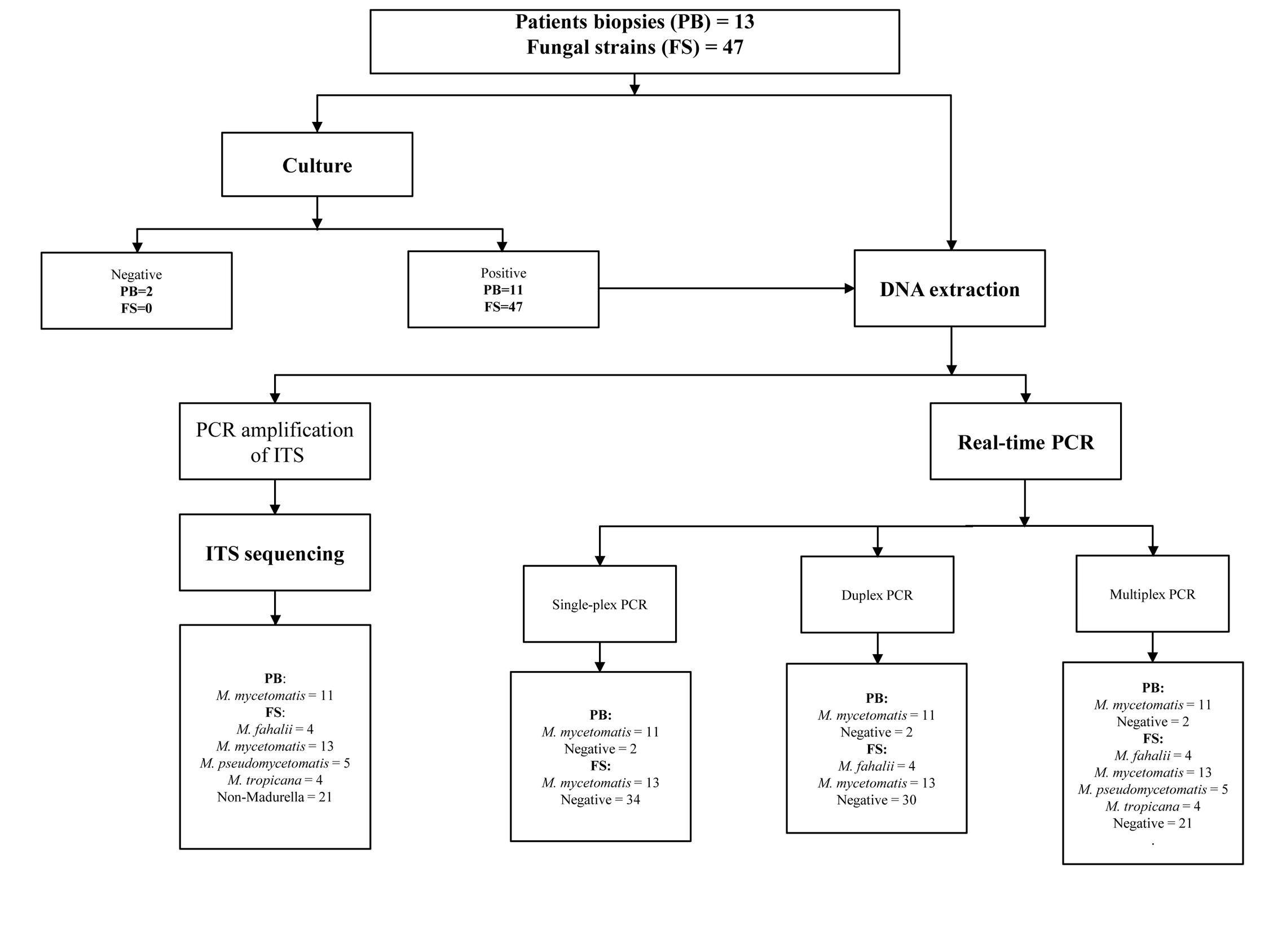

Supplement: S2 Fig — (TIF) [file pntd.0007845.s002.tif]
